# Comparative Evaluation of Oxidative Stress Modulating and DNA Protective Activities of Aqueous and Methanolic Extracts of *Acacia catechu*

**DOI:** 10.3390/medicines4030065

**Published:** 2017-09-05

**Authors:** Ashwini Patil, Manisha Modak

**Affiliations:** 1 Department of Biotechnology, Viva College, Mumbai 401303, Maharashtra, India; sfurti1012@gmail.com; 2 Department of Zoology, Sir Parashurambhau College, Pune 411030, Maharashtra, India

**Keywords:** *Acacia catechu*, aqueous extract and oxidative stress modulating activity, oxidative stress, in vitro assays for oxidative radical scavenging activity

## Abstract

**Background:** Plant-derived bioactive compounds are becoming immensely important as potential drugs. Different solvents are being used for extraction of these phytochemicals. Evaluation of biological activities of aqueous plant extracts is important as water soluble compounds would be more beneficial with respect to certification, safety and commercial issues. Oxidative stress is involved in development of many diseases; therefore, antioxidants are now being looked upon as convincing therapeutics against such diseases. Natural antioxidants are in high demand because of their lesser side effects. This study aims to compare the antioxidant activity of aqueous and methanolic extracts of *Acacia catechu*—a traditional medicinal plant. **Methods:** The activity was examined using different in vitro systems including radical scavenging activity, lipid peroxidation and inhibition of ^•^OH radical induced DNA damage using standard protocols. **Results:** Both aqueous and methanolic extracts of *Acacia catechu* show significant activities with no differences in the efficacies of water and methanol soluble bioactive compounds. **Conclusions:** Present study revealed that aqueous extract of *A. catechu* has equal potential to be used as antioxidants as compared to methanolic extract. This can contribute to increased demand of physiologically compatible bioactive compounds of natural origin.

## 1. Introduction

Medicinal plants are currently of considerable importance because of their phytochemicals which has potential therapeutic value that lead them to the path of development of novel drugs. These compounds have a wide range of biological effects including bacteriocidal, antinflammatory, antiallergic, hepatoprotective, antithrombotic, antiviral, anticarcinogenic, cardioprotective, etc. [1]. Such plants are alternative medicines for treatment of various diseases due to their assumed acceptability, effectiveness, affordability, safety and low cost [2]. Presence of beneficial phytochemicals and the shift towards natural products in pharmaceutical and cosmeceutical industry made medicinal plant research equally important to conventional drug. 

Extraction and separation of effective pharmaceuticals, looking for leading compounds, is the first and most important step in the new drug development. Numerous methods like conventional solvent extraction, steam distillation, and sublimation, etc., are developed for extraction of phytochemicals. However, these methods are based on sequential extraction, including one or more organic solvents. Such phytochemical extracts need to be processed for the removal of traces of the organic solvents. Furthermore, the mixture has to be purified for individual phytochemicals. While such methods are useful for extraction and purification of small quantities of phytochemicals for research purposes, completely removing the organic solvents from the extracts is a problematic issue [3]. Furthermore, the types and concentrations of organic solvents must be carefully selected to avoid structural changes to the target phytochemicals during extraction. Such changes adversely affect one or more of their desirable physical, chemical, and biological properties. Water, is an inexpensive, environment- friendly and an ideal solvent for the industrial extraction of medicinal plants, but its use is limited due to poor extraction efficiency for most organic compounds. New methods including water as a solvent, like superficial fluid extraction, subcritical water extractions are evolving for medicinal plants so it would be interesting to compare the bioactivities of water extracts with standard alcohol extract [3].

Phenolics and flavonoids are the major bioactive compounds found in plants. It is reported that the antioxidant capacity of a plant is mainly because of their phenolic and flavonoid content [4]. Research in medicinal plants is focused on antioxidant potential because oxidative stress has been implicated in various pathological conditions including cardiovascular diseases, cancer, neurological disorders, diabetes, ischemia/reperfusion and ageing [5,6,7]. Exogenous chemical and endogenous metabolic processes in cell produce reactive oxygen species (ROS). These are capable of oxidizing biomolecules and generate cellular oxidative stress i.e., imbalance between formation and neutralization of prooxidant. Antioxidant defense mechanisms of the cell attempt to scavenge these free radicals before causing damage. The damaged molecules are repaired as in case of DNA or replaced like in most oxidized proteins and lipids. Intracellular antioxidant defense mechanisms include antioxidant enzymes like calatalse, superoxide dismutase, etc. and, glutathione, uric acid like antioxidant molecules which scavenge the free radicals. Antioxidants are potential pharmacological agents for the treatment of many diseases. Several antioxidant rich herbal preparations are already in use for years to treat variety of diseases for their natural origin and less side effects. The pharmacological relevance of these plants are due to their secondary metabolites which are accumulated in various parts of the plant [8,9]. Antioxidants have a characteristic feature of alteration of redox state of the cell by free radical scavenging. Because of this characteristic, antioxidants has been proved to be an effective therapeutic option as reported in case of neurodegenerative diseases [10]. Current research on free radicals has highlighted the importance of antioxidant rich foods in prevention of cardiovascular diseases and cancer [6]. 

India is the largest producer of medicinal herbs and is called the botanical garden of the world. Several medicinal plants, traditionally used for over 1000 years named *rasayana* are present in herbal preparations of Indian traditional health care systems [11]. Many authors reported that it is necessary to establish a correlation between chemical and biological properties and the therapeutic activities in traditional medicine [12]. 

*Acacia catechu (A. catechu)*, commonly known as “khair” in India has been used since ancient times in ayurvedic medicine [7]. The chief constituents of the red heartwood are epictechin [13], catechin [14] and catechu tannic acid along with small proportion of brown coloring matter. It also contains tannin, flavotannin, gallotannin, phloratannin [14] epicatechin-3-*O*-gallate, epigallocatechin-3-*O*-gallate, quercetin, (+)-cyanidanol [7]. The mucilaginous gum exudates from the tree are also reported to have antioxidant activity. The heartwood extract of *A. catechu*, called pale catechu or ‘katha’ in Hindi, is a traditional ayurvedic medicine used in the treatment of cough, dysentery, throat infections, chronic ulcers and wound. *A. catechu* is also reported to possess antimicrobial [15], anti-inflammatory, antipyretic, antidiarrhoeal, hypoglycemic and hepatoprotective properties [16]. *A catechu* contains epicatechin which is claimed to be responsible for regeneration of pancreatic β cells [13]. It increases cAMP levels of the islets, which results in increased insulin release. Above mentioned pharmacological activities are due to presence of polyphenolic compounds. These compounds can alter signaling pathways and gene expression because of their free radical scavenging and metal chelating properties [17].

Current isolation and chemical purification methods used for bioactive compounds include solvent extraction processes that utilize solvent polarity as a major separation technique. These methods frequently include the use of ethyl acetate, phenol/chloroform, water, and several other approaches for extraction [3]. Aqueous solubility and bioactivity of a compound is a key parameter for designing and development of its drug potential.

The aim of the present investigation is to study and compare the antioxidant and oxidative DNA damage protective activities of both aqueous and methanolic extracts of one of the traditionally used medicinal plant of India, *A. catechu* (L.f.) Willd. (Family Fabaceae). The antioxidant potential of methanolic and aqueous extracts of *A. catechu* are tested by various in vitro assays, such as total phenolic content, total flavonoid content, DPPH, FRAP, ABTS, superoxide radical scavenging assay, TBARS assay, etc. Free radicals are known to interact with DNA and cause strand breaks so the ability of extracts to scavenge the free radicals and protect the DNA has also been studied.

## 2. Materials and Methods

### 2.1. Extract Preparation

Authentic *A. catechu* bark was procured from Dapoli Krishi Vidyapeeth, Dapoli. The solid slabs were finely powdered and 10 g of it was mixed with 100 mL water and methanol separately to prepare aqueous and methanolic extracts. Mixture was kept on magnetic stirrer for 1 h and boiled for 30 min. It was then centrifuged for 10 min at 4 °C. The supernatant was collected and aliquots stored in vials. This was considered as 100% aqueous and methanolic extract. The prepared extracts were stored at −20 °C until use. At the time of use, the required dilutions were made with water for both the extracts

### 2.2. Determination of Phenolic Content

The total phenolic content was estimated using standard protocol [18]. The measurements were compared with a standard curve of gallic acid and expressed as mg of gallic acid equivalents per g powder. 

### 2.3. Determination of Flavonoid Content

The total flavonoid content was estimated using standard protocol [19]. A standard graph using riboflavin was plotted and expressed as mg of riboflavin equivalents per g powder. 

### 2.4. 2,2-Diphenyl-1-Picrylhydrazyl (DPPH) Radical Scavenging Assay

DPPH radical scavenging assay was performed using standard protocol [20]. A calibration curve was prepared using ascorbic acid as a standard. Radical scavenging capacity of extract was measured by calculating percentage of DPPH radical scavenged.

### 2.5. Ferric Reducing Antioxidant Power (FRAP) Assay

FRAP assay was performed by using standard protocol [21]. A calibration curve was prepared using ferrous sulphate standards. Ferric reducing antioxidant power of the extracts was calculated by comparing with the standards. Results were expressed as μmol equivalents of Fe (II) per mg of powder (FRAP value). 

### 2.6. 2,2′-Azinobis-(3-Ethylbenzothiazoline-6-Sulfonic Acid) (ABTS^•+^) Radical Scavenging Assay

ABTS^•+^ radical scavenging activity was accomplished by using standard protocols [22]. A calibration curve was prepared using ascorbic acid standards. ABTS^•+^ radical scavenging activity of the extracts was measured by comparing the decrease in absorbance with standards. Results were expressed as % ABTS^•+^ scavenged.

### 2.7. Superoxide Radical Scavenging Assay

Standard protocol was used to estimate superoxide radical scavenging activity [23]. A calibration curve was prepared using ascorbic acid as a standard. Radical scavenging capacity of extract was measured by calculating percentage of superoxide radicals scavenged. 

### 2.8. Inhibition of Lipid Peroxidation

Mitochondria were isolated from goat liver using standard protocol and confirmed by estimating succinate dehydrogenase activity using standard protocol [24]. For measurement of lipid peroxidation ascorbate-Fe^2+^ system was used to induce oxidative damage to mitochondria. The concentrations of Ascorbate, Fe^2+^ and mitochondrial extract used were standardized to get optimum amount of damage. The reaction mixture was incubated at 37 °C for 25 min. The treated mitochondria were boiled with 500 μL TBA reagent in boiling water bath for 20 min and immediately cooled on ice. The pink color produced was measured at 532 nm. Inhibition of lipid peroxidation was calculated. Protection of damage was tested by adding different concentrations of extracts in the reaction mixture. 

### 2.9. DNA Protection Assay

The DNA damage protective activity of *A. catechu* extracts were performed using the protocol of Zhao et al. [25] super coiled pUC 18 DNA (Bangalore Genei). Plasmid pUC 18 was damaged by generating hydroxyl radicals by Fenton’s reaction. The linear strands formed because of the damage show band at a different position as compared to supercoiled plasmid on agarose gel. The reactions for DNA protection assay were set by adding different concentrations extracts prior to inducing DNA damage. The DNA was electrophoresed on 1% agarose gel and observed on UV- transilluminator. These were scanned using gel documentation system (Kodak, gel logic imaging system 112, Carestream Health Inc, Woodbridge, CT, USA).

### 2.10. Statistical Analysis

All experiments were performed in triplicates and repeated three times. Experimental results were mean value ± SD of three parallel measurements. All statistical analysis was conducted using Microsoft Excel. Differences among treatments were determined using student’s *t*-test. Differences at *p* < 0.05 were considered to be significant. IC_50_ values were calculated using the graph of scavenging activity against the concentrations of the samples. IC_50_ is defined as the amount of antioxidant necessary to decrease the initial radical by 50%.

## 3. Results

### 3.1. Determination of Phenolic Content

**Figure 1:**
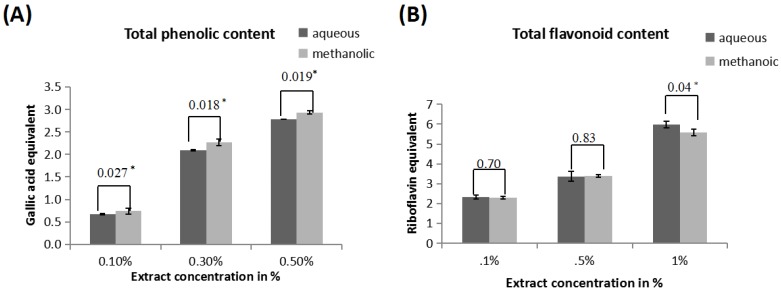
(**A**) Total phenolic contents of different concentrations of aqueous and methanolic extracts of *A. catechu* (0.1%, 0.3% and 0.5%) was measured and expressed in terms of mg of gallic acid equivalents per g of powder. (**B**) Total flavonoid content of different concentrations of aqueous and methanolic extracts of *A. catechu* (0.1%, 0. 5% and 1%) was measured and expressed in terms of mg of riboflavin equivalents per g powder. * Statistically significant (*p* ≤ 0.05).

The total phenolic content was estimated from gallic acid standard curve and evaluated as equivalent of gallic acid and expressed as mg/g of fresh weight. Figure 1A indicates the gallic acid equivalent values for 0.1%, 0.3%, 0.5% aqueous and methanolic extracts. The values for aqueous extract are 0.66 ± 0.015, 2.09 ± 0.016, 2.78 ± 0.005 respectively and for methanolic extract are 0.74 ± 0.068, 2.27 ± 0.071, 2.93 ± 0.041. As concentration of extracts increases, phenolic content also increases; 0.3% and 0.5% methanolic extracts show significantly higher (*p* ≤ 0.05) phenolic content than aqueous extract.

### 3.2. Determination of Flavonoid Content

The flavonoid content was calculated from riboflavin standard curve and estimated as riboflavin equivalent and expressed as mg/g of powder Figure 1B indicates that the total flavonoid content (equivalent of riboflavin) for 0.1%, 0.5%, and 1% aqueous extract are 2.33 ± 0.09, 3.37 ± 0.244, 5.99 ± 0.163 respectively and for 0.1%, 0.5%, 1% extracts are 2.30 ± 0.063, 3.41 ± 0.065, 5.58 ± 0.177 respectively. As concentration of extract increases total flavonoid content also increases; aqueous as well as methanolic extracts show nearly similar flavonoid content (riboflavin equivalent).

### 3.3. 2,2-Diphenyl-1-Picrylhydrazyl (DPPH) Radical Scavenging Assay

**Figure 2:**
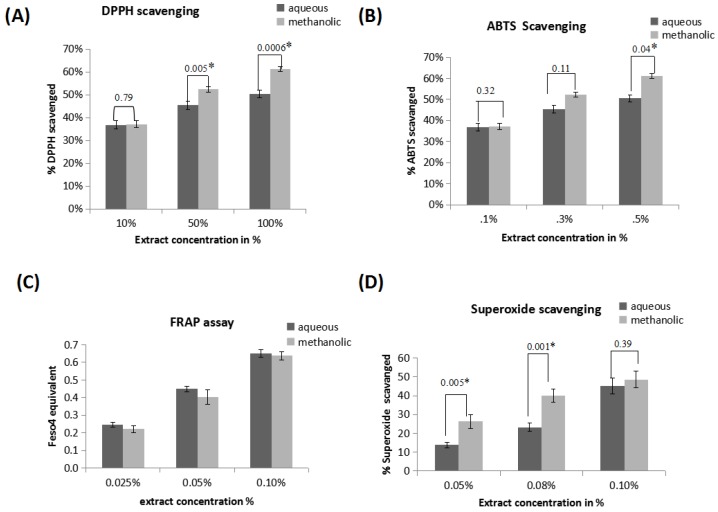
In vitro radical scavenging assays for different concentrations of aqueous and methanolic extracts of *A. catechu.* (**A**) DPPH radical scavenging activity of 10%, 50% and 100% aqueous and methanolic extracts of *A. catechu* was expressed in terms of % scavenged using ascorbic acid as a standard. (**B**) 2,2′-Azinobis-(3-ethylbenzothiazoline-6-sulfonic acid) (ABTS^•+^) radical scavenging activity of 0.1%, 0.3% and 0.5% aqueous and methanolic extracts of *A. catechu* in terms of % ABTS scavenged. (**C**) Ferric reducing antioxidant power of 0.025%, 0.05% and 0.1% aqueous and methanolic extracts of *A. catechu* in terms of FeSO_4_ equivalents. (**D**) Superoxide radical (SOD^•+^) radical scavenging assay of 0.1%, 0.3% and 0.5% aqueous and methanolic extracts of *A. catechu* in terms of ascorbic acid equivalents concentrations. * Statistically significant (*p* ≤ 0.05).

The interaction of the DPPH radical with the extract or standard ascorbic acid results in scavenging of this radical which was measured spectrophotometrically at 515 nm and expressed as % scavenging of DPPH radical. Figure 2A indicates % DPPH radicals scavenged by 10%, 50%, 100% aqueous and methanolic extract. The values for aqueous extracts are 36.75 ± 0.018%, 45.38± 0.019%, 50.48 ± 0.016% respectively and for methanolic extracts are 37.14 ± 0.016%, 52.43 ± 0.012%, 61.19 ± 0.011% respectively. As extract concentration increases % DPPH scavenging activity also increases. 50% and 100% methanolic extracts show significantly higher (*p* ≤ 0.05) scavenging activity than aqueous extract.

### 3.4. 2,2′-Azinobis-(3-Ethylbenzothiazoline-6-Sulfonic Acid) (ABTS^•+^) Radical Scavenging Assay

The total antioxidant capacity of the various concentrations of extracts was evaluated based on the decrease in colour of ABTS^•+^ which was quantified at 734 nm. The interaction of the ABTS^•+^ with the extract or standard ascorbic acid results in scavenging of this cation which is expressed as % ABTS^•+^ scavenging activity. Figure 2B indicates the % ABTS^•+^ scavenging activity values of aqueous and methanolic extracts. The % ABTS^•+^ scavenging activity for 0.1%, 0.3%, 0.5% aqueous extract are 17.60 ± 0.033, 39.38 ± 0.041, 62 ± 0.022 and for methanolic extract are 21.86 ± 0.051, 44.69 ± 0.022, 66.69 ± 0.017. As extract concentration increases, the % ABTS^•+^ scavenging activity also increases; methanolic extract shows higher scavenging activity than aqueous extract which is statistically significant (*p* ≤ 0.05) only at 0.5%.

### 3.5. Ferric Reducing Antioxidant Power (FRAP) Assay

The ferric reducing capacity of various concentrations of extracts was evaluated based on comparison of the colored product formation with standard FeSO_4_, which was measured spectrophotometrically at 595 nm. The interaction of the extracts or standard with FeSO_4_ results in reduction of FRAP reagent. The ferric reducing capacity of extracts is estimated in equivalent standard and expressed as μmol of Fe(II)/mg of powder. Figure 2B indicates the FRAP values of aqueous and methanolic extracts. FRAP values for 0.025%, 0.05%, 0.1% aqueous extract are 0.246 ±0.014, 0.449 ± 0.016, 0.653 ±0.022 and for methanolic extract are 0.221 ± 0.018, 0.404 ± 0.042, 0.638 ± 0.024. As extract concentration increases the ferric reducing capacity also increases; aqueous extract shows slightly higher ferric reducing activity than methanolic extract.

### 3.6. Superoxide Radical Scavenging Assay

The decrease of the absorbance at 560 nm with the plant extracts or standard ascorbic acid indicates their ability to quench superoxide radicals from reaction mixture and expressed as % radical scavenged. Figure 2D indicates the % superoxide radical scavenging values for 0.05%, 0.075%, 0.1% aqueous extract are 13.79 ± 1.41%, 23.23 ± 2.15%, 45.19 ± 4.11% respectively and for methanolic extract are 26.32 ± 3.76%, 40.01 ± 3.41%, 48.49 ± 4.37% respectively. As concentration of extract increases the % superoxide radical scavenged also increases; methanolic extract shows higher activity than aqueous extract which is significant (*p* ≤ 0.05) at 0.05% and 0.075%.

### 3.7. Inhibition of Lipid Peroxidation

**Figure 3:**
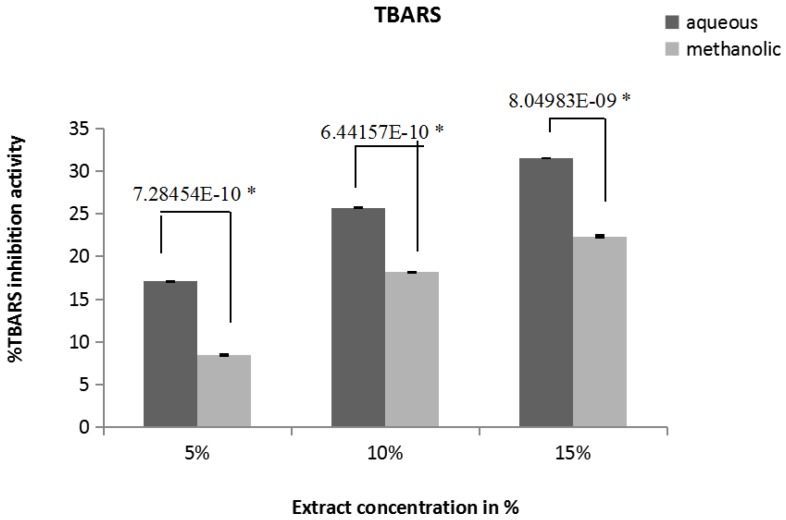
Inhibition of lipid peroxidation. Inhibition of lipid peroxidation measured in terms of TBARS (thiobarbituric acid reactive substances) in oxidatively damaged goat liver mitochondria by different concentrations of aqueous and methanolic extracts of *A. catechu* (5%, 10%, 15%). * Statistically significant (*p* ≤ 0.05).

The inhibition of lipid peroxidation activity of extract was determined by quantification of thiobarbituric acid-reactive substances (TBARS). The TBARS generated, were spectrophotometrically measured at 532 nm. Figure 3 indicates the % thiobarbituric acid-reactive substances (TBARS) inhibition capacity values for 5%, 10%, 15% aqueous extract are 17.08 ± 0.034%, 25.73 ± 0.04%, 31.49 ± 0.01% respectively. The values for methanolic extracts are 8.45 ± 0.036%, 18.15 ± 0.009%, 22.34 ± 0.095% respectively. As extract concentration increases, % TBARS inhibition activity also increases; aqueous extract shows significantly higher (*p* ≤ 0.05) TBARS inhibition activity than methanolic activity. 

### 3.8. DNA Damage—Protection Assay

**Figure 4:**
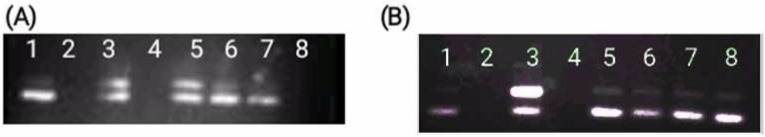
Protection to plasmid DNA pUC18 against oxidative damage. Gel electrophoresis pattern of pUC18 plasmid DNA after induction of oxidative damage in the presence or absence of different concentrations of aqueous and methanolic extracts of *A. catechu*. (**A**) Oxidatively damaged DNA in presence of aqueous extracts of *Acacia catechu* Lane 1: control pUC18 DNA, lane 3: oxidatively damaged DNA; lane 5: oxidatively damaged DNA in the presence of 0.01% extract, lane 6: oxidatively damaged DNA in the presence of 0.05% extract, lane 7: oxidatively damaged DNA in the presence of 0.1% extract. (**B**) Oxidatively damaged DNA in presence of methanolic extracts of *A. catechu* Lane 1: control pUC18 DNA, lane 3: oxidatively damaged DNA; lane 5: oxidatively damaged DNA in the presence of 0.01% extract, lanes 6: oxidatively damaged DNA in the presence of 0.05% extract, lane 7: oxidatively damaged DNA in the presence of 0.1% extract, lanes 8: oxidatively damaged DNA in the presence of 0.5% extract.

The ROS mediated DNA damage was induced using methods of Zhao et al. [25]. DNA was damaged by OH^∂^ formed in fenton reaction. Figure 4A,B indicates the concentration dependent DNA protection potential in methanolic and aqueous extracts of *A. catechu*. As the concentration of extract increases intensity supercoiled band increases as compared to damaged one. 

## 4. Discussion

In the living system, free radicals are constantly generated and they can cause extensive damage to tissues and biomolecules. This leads to various disease conditions, especially degenerative diseases [26] like aging, cancer, cardiovascular disorders, cataracts [27] neurodegenerative diseases such as Alzheimer’s disease, Parkinson’s disease, and amyotrophic lateral sclerosis [28]. Therefore, antioxidant research is important in medical field and food industry and has received much attention. The body possesses defense mechanisms, in the form of antioxidant enzymes and molecules, which minimize the damage caused by ROS. Even then continuous exposure to chemicals and contaminants can increase in the number of free radicals in the body beyond the natural capacity of the body to control them causing irreversible oxidative damage [29]. 

A growing human population, global climate change and the change of terrestrial food resources for energy needs in recent times have raised serious global food security concerns. There has been a quest to explore and use foods from diverse sources and to enhance and supplement the nutritional quality of human foods. Antioxidant molecules are gaining lot of interest in this respect. Antioxidants are also attracting the attention of the scientific community because of their potential to be used as a dug and supplement to drug in case of many degenerative diseases to minimize the complications. Synthetic antioxidant like butylatedhydroxy anisole (BHA) or butylatedhydroxy toluene (BHT) or propyl gallate (PG) are supplemented to reduce the oxidative damage but due to side effect these are not well accepted antioxidant [30] Therefore, attention is directed to find out the alternative remedy from non-toxic, natural resources with strong antioxidant properties. Thus, natural antioxidants are in high demand for application as supplement to food, drug, etc., Plants with their compounds, like phenolics and flavonoids have been reported to have multiple biological effects, including antioxidant activity [31,32]. It is generally assumed that frequent consumption of plant-derived phytochemicals from vegetables, fruit, tea and herbs may contribute to shift of balance towards an adequate antioxidant status [33]. The extraction of these bioactive phytochemicals uses organic solvents but the organic solvents lead to the issue of safety and acceptability by human body. Organic solvents may also change the molecular structure of the compound which may lead to modulation in its activity [34]. In this regard water extraction is always a better but neglected option because all phytochemicals are not extracted in water. Therefore the present study is focused on the comparison of antioxidant activity of aqueous and methanolic extract of *A. catechu*.

The phytochemical analysis showed that *A. catechu* methanolic and aqueous extract are rich in flavonoids and phenols. Our result shows that phenolic content of methanolic extract is higher than aqueous extract but flavonoid content is nearly similar in both the extract. Flavonoids exhibit their antioxidative action through scavenging or chelating process [35]. The phenolic compounds have been reported to be significantly associated with the antioxidant activity of plant and food extracts mainly because of their redox properties, allowing them to act as reducing agents, hydrogen donors, singlet oxygen quenchers, hydroxyl radical quenchers, and metal chelators [36]. The presence of both these compounds in human diet and their effects on human nutrition and health are considerable.

The antioxidant activity of herbal extracts and phytochemicals is routinely estimated using free radical scavenging assays. The ability to donate hydrogen is the key parameter of primary antioxidant. The labile hydrogen is abstracted by DPPH radical which can be interrelated with inhibition of lipid peroxidation [37]. The % DPPH radical scavenging values of the methanolic extracts are significantly higher than aqueous extracts. Based on Table 1 IC_50_ data, both aqueous and methanolic extracts do not show statistically significant difference in their radical scavenging activity.

The FRAP value reflects the reducing power of the extracts. The extract revealed the ability to reduce Fe^3+^ to Fe^2+^ [38]. From Table 1 IC_50_ data, it can be observed that both the extracts of *A. catechu* showed FRAP scavenging activity.

Lipid peroxidation is the most important consequence after oxidative damage to cell or mitochondria [39]. Malondialdehyde and other aldehydes have been identified as products of lipid peroxidation that react with thiobarbituric acid (TBA) to give a pink colored species that absorbs at 532 nm [24]. Here we have shown the inhibition of formation of TBARS by aqueous and methanolic extracts. There is also no statistically significant difference in IC_50_ values for inhibition of TBARS for both the extracts.

Superoxide anion has harmful effect on the cellular components in biological systems [40]. Superoxide radical is a potent reactive oxygen species and is highly toxic at fairly low tissue concentrations. Moreover, although this anion is a weak oxidant, it reacts with other molecules in tissues to form highly toxic hydroxyl radicals as well as single oxygen, both of which are significant contributors to oxidative stress [6]. Our IC_50_ data indicates that both the extracts show similar superoxide radical scavenging activity.

DNA is one of the major biomolecules, which is damaged by free radicals [25]. The DNA protection ability of the extracts by scavenging the free radicals was also estimated. Oxidative damage to plasmid DNA caused by free radicals generated from Fenton reaction was measured in terms of conversion of supercoiled to nicked circular form. Protection offered by the extracts was estimated in presence and absence of these compounds. The present study revealed that DNA is protected from deleterious effects of hydroxyl radicals by both the extracts. 

In this study, water was used to extract hydrophilic antioxidants which can be used in food. There is always a conflict about extraction of bioactive compounds in water, as most of them are nonpolar but *Acacia catechu* is rich in tannins, catechin and epicatechin [41] which are water soluble [41]. This justifies the comparable antioxidant activity of aqueous extract with methanolic extract of *A. catechu.* However, epicatechin as a pure molecule is believed to be a structure modifying toxin, so for preparation of pure form of medicine from *A. catechu*, the toxicity studies must be carried out. The exact molecules which are responsible for antioxidant activity of *A. catechu* must be investigated. However, the whole plant extract investigated is nontoxic to animals as well as to humans [41], so it can be a good option to be used as a medicine. Plant extracts made with water are advantages in relation to certification and safety. In this respect we demonstrate that, like methanolic extracts, aqueous extracts too have oxidative radical scavenging capacity with potential for use in various medicinal and nutritional products.

## Figures and Tables

**Table 1:** IC_50_ values for in vitro antioxidant assays. $ Unit of IC_50_ of all activities is mg of equivalents/g powder. Each value represents mean ±SD. (*n* = 9).

Assay	Aqueous Extract IC50 $	Methanolic Extract IC50 $
Total Phenolic Content	50.28 ± 0.272	50.20 ± 0.218
Total flavonoid content	49.87 ± 0.236	40.70 ± 0.482
DPPH radical scavenging	52.18 ± 0.0686	54.44 ± 0.120
FRAP	52.21 ± 0.043	52.02 ± 0.0043
ABTS^•+^ radical scavenging	50.21 ± 0.047	50.01 ± 0.0042
Superoxide radical scavenging	50.25 ± 0.399	49.74 ± 0.161
Lipid peroxidation	48.65 ± 0.103	49.65 ± 0.241
